# In the Line of Duty: Militarizing African Epidemics

**DOI:** 10.1111/1758-5899.13297

**Published:** 2023-11-16

**Authors:** Tim Allen, Melissa Parker

## Abstract

The deployment of soldiers for epidemic control in Africa has become more acceptable, even when human rights violations occur. This article outlines how this situation has arisen, foregrounding overlapping processes since the 1990s and the implications of Security Council Resolution 2177. It then explores effects with reference to Sierra Leone and Uganda. Drawing on long term fieldwork, it discusses militarised epidemic control programmes during Ebola and COVID-19 outbreaks. It points out similarities in the responses to epidemics in these two countries, including the violent enforcement of regulations, but also striking differences. In Sierra Leone, a democratic transition of governmental power occurred, whereas militarised epidemic control in Uganda helped entrench autocratic public authority. To the extent that there is data available, disease control outcomes in the two countries were not widely divergent, yet the Ugandan response has been valorised. This highlights a drift towards less accountable forms of governance, justified by purported public health objectives.

## Introduction

In October 2022, an article in Uganda’s New Vision newspaper described how five health workers were exposed to Ebola ‘in the line of duty’ (Okoth 2022). Television coverage of their discharge from hospital was revealing. They were sitting in a line, wearing identical blue overalls, surrounded by staff wearing green. It looked like they were being paraded as prisoners of war. The Ugandan Minister of Health issued them ‘discharge certificates’, as if they were being awarded amnesty documents. President Museveni, Uganda’s head of state, had proclaimed that Ebola “is an enemy we can easily fight” (Kasibwe 2022), and security rhetoric was widespread in the Ugandan media. There were references to health care workers as ‘Ebola Fighters’ (Sserugu 2022); to those avoiding monitoring as ‘escapees’, and those who might be infected as ‘suspects’ (Wanderer et al 2022). Privately, staff working for international agencies expressed concerns to us about the approach. However, they should not have been surprised. The militarization of disease control has escalated in the past decade with international support. Using a public authority lens, we discuss how the situation has come about and comment on the implications for two African countries: Uganda and Sierra Leone.

The term ‘public authority’ is used to refer to any kind of authority beyond the immediate family that commands a degree of consent (CPAID 2018). A public authority lens focuses on the relationship between formal, informal, parallel and hybrid authorities; and foregrounds socio-political dynamics that might otherwise remain hidden from view (Parker et al., 2019a; Kirk and Allen, 2021). In so doing, it provides an opportunity to explore how, and why, ostensibly similar policy measures are implemented, experienced, and perceived in different contexts. The approach proves illuminating when comparing militarised epidemic control measures in Uganda and Sierra Leone, and assessing ways in which their national responses have been linked to global policy agendas and evaluated by public health analysts.

The two countries have similarities in that they both share a history of British colonial rule; have experienced prolonged periods of insecurity, and have had Ebola and COVID-19 epidemics. They also have national armies with close connections to militaries in the UK and the USA, and they have both used their armies to enforce behavioural change during epidemics. Nevertheless, the two countries have had different political trajectories. In Sierra Leone, a democratic transition of governmental power occurred in the wake of Ebola, whereas militarised epidemic control in response to COVID-19 in Uganda helped to entrench autocratic public authority. A key point made in the article is that militarised enforcement during epidemics has been actively promoted or condoned by institutions which in other circumstances would have viewed it as involving unacceptable human rights violations. Moreover, the autocratic trajectory in Uganda has been valorised for purported public health benefits, while democratic efforts in Sierra Leone have had less attention. That is the case, even though there is little or no evidence that instrumental force has been an effective means of disease control. It is an aspect of a broader trend in response to public health crises and is likely to shape future policy choices.

To make this argument, we briefly discuss the background to the expansion of militarised public health enforcement and then draw on findings from local-level fieldwork and discussions with individuals involved in policy planning and implementation, including those working at national and international levels. Our research has been carried out over several years as part of multiple research projects (listed in the acknowledgements) and involved working with colleagues based in the two case study countries. In Sierra Leone, research took place between 2017-2020 in the southern region. This work initially focused on the socio-political dimensions of the 2014-2016 Ebola outbreak (e.g., Parker et al 2019a, 2019b), and subsequently on the legacies of militarising humanitarian assistance. In Uganda, research took place from 2004 and included a focus on different aspects of public health, justice, social healing, and governance in northern, north-western, and western Uganda (e.g., Allen 2006; Allen and Reid 2015; Allen et al 2020; 2022; Parker et al 2008; 2011; 2012). This research informed broader collaborative work, focusing on epidemic preparedness and response between 2019 and 2022 (e.g., MacGregor et al 2021; Leach et al 2022; Akello and Parker 2021; Parker et al 2020; 2022). Most of the fieldwork involved participant observation and open-ended ethnographic interviews. Details about the range of methods used are described in the cited articles, as well as in publications discussing the challenges of doing long-term research of this kind (e.g., Allen and Parker 2012; Parker and Allen 2013).

### Militarisation and Epidemics

Compulsory compliance in public health agendas is unusual in economically affluent countries. COVID-19 marked a departure in that respect. The policing of behavioural restrictions was something new for most populations, and the deployment of soldiers in Italy, Spain and elsewhere a shock. Voluntary participation in health programmes has also been a characteristic of international development schemes, even when a degree of enforcement might have been a logical step in disease control, for example with respect to HIV/AIDS, schistosomiasis and lymphatic filariasis (Allen 2006; Parker and Allen 2011, 2013). However, connections between militaries and disease control have a long history. Quarantines and behaviour changes were imposed, for example, in response to the Black Death and outbreaks of cholera, and military personnel have long taken leading roles in medical research (Briselli et al 2022; Tognotti 2013). A connection with warring armies was also integral to the establishment of the Red Cross, much to Florence Nightingale’s distain (Allen 2018). Meanwhile, in European colonies, mass forced population displacements and other violent measures were implemented by soldiers and armed police, purportedly to prevent the spread of disease (Vaughan 1991). A militarised discourse of health provision was even integrated into the new arrangements aimed at establishing a better world in the 1940s. The World Health Organization’s constitution explicitly linked the ‘health of all people’ to the attainment of ‘peace and security’ (WHO 1948, July 22). It thereby deliberately connected the new organization with Chapter VII of the UN Charter, which required the Security Council to initiate military action when necessary to ‘maintain or restore international peace and security.’

During the Cold War, divisions in the Security Council made such enforcement unlikely, but that changed in the 1990s. International and national endorsement for militarised health care escalated, and this is reflected in a growing literature (e.g., Lakoff and Collier 2008; Rushton 2019; Michaud et al 2019). Since the early 1990s four overlapping trends have been particularly important, not least because they have fed into what might be understood as a growing consensus in certain contexts about what is appropriate, or at least acceptable, during epidemics. They are trends that have effectively reshaped public authority paradigms in international relations, with profound local effects.

First, during the upheavals of the 1990s, it became impossible to sustain a distinction between humanitarian assistance, aimed as assisting people in dire need, and humanitarian intervention, aimed at forcibly preventing atrocities. For the most part, agencies like MSF vigorously opposed military involvement in health programmes, but the tensions were hard to set aside when appalling violence was occurring. Bernard Kouchner, one of the founders of the agency argued that standing aside was unacceptable, and he embraced the possibilities of enforcement as a preferable option, much to the irritation of his former colleagues. He used his position in the French government and in the UN to actively promote ‘a right to interfere’ (Allen and Styan 2000). While his ideas were controversial, by the end of the 1990s, there was widespread recognition of failures in humanitarian responses, especially those in Rwanda and former Yugoslavia. Neutral medical aid, it seemed to many, was not enough. This underpinned the emergence of Responsibility to Protect (R2P) (ICSS 2001). MSF strongly opposed the R2P agenda, arguing that it effectively justified military invasions by powerful states, but it was adopted by UN member states at the World Summit in 2005, and subsequently influenced international responses to Libya and elsewhere.

A second, closely related, development was the promotion of ‘human security’ from 1994 by the UN. This has not had the effect that was anticipated. Initially, the UN Human Development Report explicitly equated human security with people rather than territories, and with development rather than arms. However, governments have been more inclined to explore new ways of deploying soldiers, and less inclined to set aside military responses to crises. Mobilising the term ‘security’ has helped to create more space for that to occur. Thus, the approach outlined in the UN *Human Security Report: War and Peace in the 21*^*st*^ *Century* (2005), was explicitly orientated to the use of armed forces to better limit the adverse effects of violent conflict. Rather than removing arms, a discourse of human security encouraged the use of militarised enforcement in situations in which, in the past, involvement of soldiers, militia, and armed police was viewed as potentially problematic. Conceptualised as a new way of promoting development and providing services that could contribute to UN goals, human security has contributed to making militarised securitization a more acceptable aspect of policy, something that has been embraced openly by authoritarian governments. As King and Murray note, ‘economic development and military security became intertwined’ (King and Murray 2002).

Third, this apparent invitation to link militarised enforcement with health care and other services by the UN was enhanced by positive assessments of militarised policies in some states, including China. The Chinese government has openly taken pride in deploying its military for purposes of disease control, and enthusiasts of the government have extolled the achievements of the Chinese system. During the 2000s, the Chinese government passed laws and set out regulations on responses to emergent public health hazards which stipulated the involvement of the People’s Liberation Army. Subsequently, it has been claimed that Chinese military forces helped prevent epidemics of acute respiratory syndrome (SARS), influenza A (H1N1), avian influenza H5N1 and H7N9, and Ebola, while actively maintaining public health, economic development, and national construction (Ma et al 2016). Such a glowing assessment is perhaps open to question, but the draconian militarised response to COVID-19 in China has illustrated enforced epidemic control measures that governments of other states might have liked to emulate if they could have done so. Some certainly tried, including Uganda. It is also worth noting that the director-general of the World Health Organization (WHO) from 2006 to 2017, was a Chinese Canadian who nurtured the WHO’s connections with China. She openly praised the Chinese government’s handling of COVID-19, supported various Chinese initiatives, and required WHO staff to refer to Taiwan as a Chinese province (Vanderklippe 2020).

Fourth, during the 1990s, analysts drew attention to the potentially adverse consequences of globalisation for public health. Laurie Garrett (1994) and others warned about the likely spread of infectious diseases as result of multiple factors, including environmental changes, prevalent air travel, and increasingly integrated food markets. Their concerns were underscored by the emergence and rapid spread of infectious diseases such as HIV/AIDS in the 1980s, SARS in in the early 2000s, H1N1 in 2009-10, and MERS in 2012. In response, emphasis was placed on the acute need for strategies to identify, prevent, and limit infections (e.g., Heymann and Rodier, 2001; Heymann 2003; Morens et al 2004; World Health Report 2007; Elbe 2008). Much of this literature evoked links between health and security. The emphasis was on securing well-being, and associations with enforcement were not explicitly discussed. However, in the context of post-Cold War armed conflicts and efforts to promote R2P, the militarised implications of the word ‘security’ were unavoidable, and threats to health linked to threats to peace (Parker et al 2022).

These four trends were dramatically linked together in September 2014. In response to the West African Ebola outbreak, and alarm about the possible spread of the disease outside the region, the UN Security Council passed Resolution 2177. The Resolution described the Ebola outbreak as ‘a threat to international peace and security’ and legitimized enforcement action. Some scholars were critical (de Waal 2014; Du Bois et al 2015). However, others were supportive or even enthusiastic. Significantly, MSF reversed its rejection of militarised medical responses, and senior staff were among the most vocal in calling for armies to intervene (Allen 2018). This was not the first time that the UN Security Council had passed a resolution related to a disease. Fourteen years previously, Security Council Resolution 1308 had addressed HIV/AIDS. That had also referred to the Council’s responsibility to maintain international peace and security but did not legitimise enforcement action. With Resolution 2177, Permanent Members of the UN Security Council agreed that soldiers were needed on the ground. It was a turning point in expanding the legitimisation of militarised enforcement to contain epidemics. After the West African Ebola outbreak, soldiers became involved in COVID-19 testing, the roll out of vaccinations, monitoring infection, and mandatory quarantine. In countries receiving international aid in Africa the shift was less of a surprise than in Europe, due partly to the trends in externally funded and directed public health norms since the 1990s, as well as awareness of past control measures for sleeping sickness and other disease during the colonial era. However, this does not mean that military deployments were necessarily welcome. The following sections discuss these issues with reference to Sierra Leone and Uganda.

### Sierra Leone

Following the Security Council Resolution, more than 5,000 military personnel were deployed to West Africa by the UK, France, the USA, Canada, and Germany. In terms of disease control, research suggests the effects were mixed (e.g., Kamradt-Scott et al 2015); and on the ground views about the activities of foreign soldiers varied widely. Predictably, the most positive accounts were provided by the militaries themselves, and these perspectives have found their way into ostensibly objective assessments. Indeed, the idea that the foreign forces were a ‘game changer’ has become received wisdom in some quarters. For example, one website linked to the Imperial War Museum in London is glowing about the role of the British army in Sierra Leone. It describes how military medics were sent to treat healthcare workers and staff tackling the outbreak and to offer training and provide security (IWM no date). Other assessments noted that militaries deployed from high income countries were often risk averse, remained in big cities (or off the coast in ships), and slow to establish Ebola Treatment Units when they attempted to do so. Nevertheless, their connection with national forces is claimed to have been a crucial aspect of disease control; and it is argued that militaries established an orderly response to management of the outbreak. In the case of Sierra Leone, Dwyer and Gbla (2021) have argued that the military deployment dramatically shifted the reputation of the Republic of Sierra Leone Armed Forces (RSLAF) away from being associated with abuses during the civil war.

By contrast, research focusing on local level responses to the Ebola outbreak tells a rather different story. Richards (2016) and Wilkinson and Fairhead (2017), for example, have argued that paramount chiefs were often the key figures exercising public authority and shaped local responses to the epidemic. The role of soldiers in their accounts is limited. However, it has been noted that where soldiers were able to act without constraint, or were encouraged to act aggressively, their approach to disease control was perceived as oppressive (Parker et al 2019a). Even the generally positive analysis of military involvement by Kamradt-Scott et al (2016) has drawn attention to instances of violence by national armed forces in containment leading to intimidation and fear. Their respondents in both Sierra Leone and Liberia noted that they followed instructions from their national armed forces out of fear for personal safety and the safety of their loved ones, and that it was not uncommon for respondents to recall memories of the civil war and past atrocities committed by both armed forces and ‘sobels’ (soldier-rebels) when discussing the role of the military in the Ebola response. Questions were also asked about why military personnel carried guns and were dressed in combat fatigues when they were ‘fighting’ a disease. Our own research in Sierra Leone focussed on experiences of Ebola control and amplifies these points about the population’s concerns about RSLAF enforcement.

In December 2014, for example, RSLAF soldiers arrived in a village in Moyamba district, (Parker et al 2019a). It was suspected that people had secretly buried loved ones who had died of Ebola. They ordered people to come out of their houses and threatened to beat them if they did not reveal where the bodies had been buried. Women described how they were thrown to the ground and kicked through the village, and men described how they were hit with the butts of guns (with one man displaying his injuries). In another location, closer to the coast, a militarised lockdown and enforced testing was so feared that a group tried to escape by boat. The boat capsized and more than twenty people were reported to have drowned, mainly women and children.

In spite of these abuses, many of which were widely known at the time, people continued to try and bypass or subvert the enforcement measures, sometimes successfully. In the village mentioned above, a secret society continued to operate, hiding infected relatives from army surveillance, treating infected relatives with rehydration remedies in the surrounding forest, and burying them in hidden graves if they died. They even created their own personal protection equipment out of empty rice sacks and plastic bags. To the extent that solders made things happen, it was due to fear and instrumental violence, and those that resisted were valorised as standing up for their loved ones.

Where possible, contacts with the army were also mobilised to help assist them. In the location where people had tried to escape by boat, a young woman with a baby realised that she was falling ill, and she rang her mother to ask her to save her grand-daughter. The house was quarantined by the army, but a small girl ducked under the barriers in the middle of the night and retrieved the child. The baby then developed Ebola symptoms and was taken by an ambulance together with her mother to a treatment centre. Sadly, they both died. Soon afterwards, the grandmother developed symptoms of Ebola. Convinced that she would die if she was taken to the treatment centre, she rang her elder daughter who was married to a soldier. He explained that she was more likely to receive effective treatment if she could reach a military hospital near Freetown. She consumed paracetamol to keep her temperature artificially low and managed to talk her way through multiple army check points. On reaching the hospital, she insisted on being admitted, where she was eventually diagnosed as positive for Ebola. She survived.

It is not known how widespread these kinds of practices were across the country, but it is reasonable to conclude from available evidence that while the overall effects of militarised epidemic control are hard to assess, they were much less significant than has been claimed. Arguably, however, there were other positive consequences. In Sierra Leone, military personnel bypassed some existing institutions and individuals exercising public authority but worked closely with others. The latter included paramount chiefs in some locations, whose powers were officially reactivated by the President. Moreover, while the deployment of RSLAF officers to districts, where they set aside existing administration and took controlling influence, provided a model for an alternative approach to organisation - it did not lead to a permanent co-option of power. Indeed, it is reported in some locations to have left a legacy of better district coordination, or a least the idea that it was possible. When the epidemic ended, the soldiers were withdrawn. Moreover, they did not intervene in the Presidential elections that took place in 2018. The incumbent President, Ernest Bai Koroma, did not stand, ostensibly because he had served the constitutionally eligible ten years in office. Yet, posters had been produced equating him with the Messiah. In his hometown, Kamabai, a slogan next to his picture still states: “This is our son with whom we are well pleased.” Nevertheless, Koroma did not use, or was unable to use, his close connections with senior army commanders to extend his period in office. After a hotly contested election, the opposition party candidate, Julious Maada Bio was declared the winner. In other words, Koroma’s handling of the epidemic did not keep his party in power, and the deployment of the army did not enhance his party’s hold on power.

Moreover, the army did not prevent the political transition, although it is important to mention that Bio is a former senior soldier. He was involved in a military coup in 1992 and led another coup in 1996. Arguably, the democratic transition of power in 2018 could be seen as a faction within the military using administrative roles they had taken on during the Ebola outbreak, with international support, to secure power. Here, it is interesting to note that a year after Bio’s new government took over, he removed some senior military figures linked to the previous government and had the opportunity to entrench his authority by taking a militarised approach to COVID-19. However, resentments in parts of the country to what had been experienced as oppressive acts by soldiers during the Ebola outbreak, and the fact that the ruling party had not been able to secure a victory in the 2018 election, were factors that help to explain the adoption of a more subtle approach to militarised disease control. Initially, there was concern that the virus would cause significant mortality and morbidity across the country. In January/February 2020, we were doing research on the aftermath of Ebola in Sierra Leone and found that military officers were more interested in asking us about COVID-19 than discussing the legacies of the outbreak. They were receiving briefings about the disease on their smartphones and anticipated being involved in lockdown measures. Interestingly, chiefs and district officials also received communications about the disease, but some days later. Indications that the Ebola model might be repeated were underlined by the fact that the Minister of Defence was assigned as the Interim Coordinator of the National COVID-19 Emergency Response Centre. Soldiers were deployed to work with the Compliance Enforcement Mechanism System which enforced preventative measures in public spaces, and additional troops were sent to border regions and other locations to restrict movement.

However, this militarised response to COVID-19 control was not openly part of a broader process of militarised governance, and once it became apparent that morbidity and mortality associated with COVID-19 was not going to replicate Ebola, the role of the military changed. A decision was made not to enforce quarantine in the way that had occurred a few years earlier. Instead, it was agreed that it would be better to use ‘psychosocial’ measures to encourage people to move to treatment centres when they were infected. There was also less international interest, and less concern by affluent countries that their populations would be exposed to infection from Sierra Leone. As one senior officer explained to Dwyer and Gbla in August 2020, “most of the countries that were major funders of the Ebola response are now themselves victims of COVID-19, struggling to address the carnage in their own backyards” (Dwyer and Gbla 2020: 29).

By mid 2021, the COVID-19 response in Sierra Leone became a reasonably well organised communication campaign, linked to containment and treatment, with the army remaining mostly in the background. As vaccination rollout was stepped up, the Ministry of Health officially drove the agenda. Militarised support was restricted to the provision of treatment in military hospitals. In November 2021, the head of the Expanded Programme on Immunisation explained what he viewed as the success of his operation. “Collaborative leadership,” he claimed, “is key to responding to a pandemic. This is the approach we took to tackle Ebola and it is the same response we have taken in the fight against COVID-19,” (quoted by Bilkis, 2021:1). In fact, COVID-19 control was very different to Ebola control for many of those on the receiving end of government policies, and notably less violent.

Overall, the army’s disengagement from politics after the Ebola epidemic and at the time of the elections has been surprising, given what has happened elsewhere. The relatively de-politicalised professionalism of the military engagement with COVID-19 has had the effect of raising RSLAF’s international reputation in some quarters; and the enhancement of the RSLAF’s public authority in the wake of Ebola is potentially enhancing formalised democratic politics. When interviewed, army officers tended to complain that the military were having to grapple with expectations that they would be more involved in domestic affairs than senior commanders considered appropriate. However, politicised divisions within the army remain. When, in August 2022, riots broke out in Freetown and the north of the country in response to the rapidly rising cost of living, a nationwide curfew was imposed. Bio removed three generals from the north, and replaced them with people from the southeastern part of the country. Arguably, it is Bio’s continued capacity to maintain his authority in the military, combined with the military’s increased emphasis on professionalism, that is allowing him to survive in a very difficult economic context, and to win re-election in 2023. The opposition have complained vehemently about vote-rigging, but a coup seems unlikely.

The contrast with Uganda is striking - not least with the way international actors have complied with the exercise of public authority by the two governments and have perceived the results. While the role of militaries in controlling Ebola in Sierra Leone has tended to exaggerate the role of international armed forces and their national counterparts, assessments of COVID-19 control in the country are muted. The army’s low-key role has barely been noticed, and overall assessments of what occurred have focussed on a few observations about access to health care. Sevalie et al (2021), for example, noted that there was a decrease in hospital use during COVID-19, but the decrease was less than that being reported in other countries during COVID-19 and less than that reported during the Sierra Leone Ebola outbreak. Although under-reporting was likely to have been high, it is interesting to note that overall COVID-related deaths reported in Sierra Leone by WHO is just 125, compared with 3,956 from Ebola. By July 2023, over 9 million COVID-19 vaccine doses had reportedly been administered to a total population of around 8.4 million. Available data are not much more revealing for Uganda, but it is not health data that has driven overall evaluations.

### Uganda

Uganda had an Ebola outbreak in October 2000. There was a total of 425 confirmed infections, with the last case occurring in mid-January 2001 (Omaswa et al 2015). The outbreak occurred in the war zone of the north, and the army were inevitably involved. It was not considered a threat to international peace and security, and there was no international intervention as later occurred in Sierra Leone. The affected region was already highly securitised, and most of the population were living in internal displacement camps. Movement was limited and aggressively controlled. Human rights violations, including killings and severe beatings, had been occurring for decades, with the Uganda People’s Defence Force (UPDF) being implicated in some of these incidents. Unlike Sierra Leone, infected people made their way or were taken to hospitals, especially the Catholic hospital at Lacor, Gulu (McPake et al, 2015). The fatality rate of those with confirmed infection was over 50 percent, but concerted efforts were made to nurse the infected. Many medical staff caught the disease and died, but experience was gained in rehydrating patients. Years later, Ugandan soldiers were sent to West Africa in 2014. They helped show Sierra Leoneans how this treatment could be implemented safely (Parker et al 2022).

Alarming accounts of the Ebola outbreak in Uganda, and its rapid containment with the involvement of the UPDF, were factors known to those calling for a military response to Ebola in West Africa. The 2000/2001 Ugandan outbreak highlighted the virulence of the disease, the vulnerability of medical practitioners, and the possible need for enforcement measures implemented by soldiers. The fact that wars were not ongoing in West Africa and therefore movement of people was not restricted by the military, made the situation appear very serious, not least because of West Africa’s proximity to Europe, and the frequency of visits with relatives living in the Global North. The Ugandan outbreak might be viewed as a kind of model for the interventions that occurred after Security Council Resolution 2177 in Sierra Leone, Liberia, and Guinea. It also informed Ugandan responses to COVID-19 in 2020-2021.

When WHO declared COVID-19 to be a Public Health Emergency of International Concern in early 2020 there were no reported cases of the virus in the country. Nevertheless, President Museveni responded quickly, and immediately militarized the national response (Parker et al 2020). A COVID-19 National Task Force with a command-and-control system was established. A lockdown was enforced by the UPDF, with additional support coming from paramilitary Local Defence Unit’s (LDUs). The military were ordered to limit movement across international borders; check that people had permission to travel; close markets, schools, mosques, churches, and other public places; ensure quarantine occurred at designated centres; check that burials, weddings and homestead gatherings were carried out in accordance with official standard operating procedures; and impose curfews (Parker et al 2022). The President explained that “This is a war of the *wanachi* [people]. I’m here to lead the *wanachi* war …”. He also warned that anyone resisting would be treated with “iron gloves deserving of enemies” (March 30^th^ 2020). When opposition politicians tried to distribute food to people who had lost their source of income during lockdown, Museveni responded by saying: “I direct police to arrest people who will be distributing food to people. That is looking for cheap popularity… you will be charged with attempted murder” (Daily Monitor, 2020).

Accounts of soldiers beating people to enforce controls in urban locations quickly started to appear in the media, and the bad publicity prompted Museveni to urge the UPDF to stop assaulting civilians in April 2020 (Hayden 2020). However, such practices continued, especially in places away from scrutiny by journalists and human rights activists. In locations in which we have been working, located in the north-western and western parts of the country close to international borders, for example, instrumental violence ostensibly for disease control purposes was normalised and incorporated into public authority arrangements. Local government officials and local councils worked closely with soldiers to institutionalise control measures, many of which were still in place two years later.

At one site, located on the northern shores of Lake Albert, the UPDF arrived at a busy market within hours of the lockdown announcement. They fired live rounds of ammunition into the air and beat traders. Over the coming weeks, restrictions were enforced by soldiers, who worked alongside LDU’s, immigration officials, police, and an armed marine security unit. Numerous people reported being attacked. A pastor, for example, who was returning from a prayer group was stopped on his motorbike and beaten until he lost consciousness. Fishermen retuning to shore in boats were especially vulnerable. As one reported, “a few minutes to the curfew time, armed men were regularly seen heading to landing sites and they intercepted whoever came late… Upon arrest, victims would be forced to remove their shirts, caned, and asked to pay fines or taken to police cells…As time went on, this became a real business for the men in uniform.”

Meanwhile, at another research site in the west, where the UPDF have had a long-standing presence monitoring the international border, soldiers were already a feared presence. The majority of people avoided contact with them. However, that became impossible once lockdown started. Beatings became common for breaking regulations, such as crossing the border or carrying passengers on the back of a motorcycle. A young man, observed: “These soldiers have no mercy…they beat you like you are a snake.”

These events occurred when there were no reported cases of COVID-19 in the area, and they were generally viewed as abusive efforts to extend the government’s power, or to increase income and enhance the authority of locally based army officers. Initially there was fear of the disease, because of accounts about deaths from COVID-19 in rich countries, but over time scepticism increased. As a schoolteacher at the site near Lake Albert observed, “Corona is considered a political disease because of the way security personnel were selective in enforcing guidelines.” Along similar lines, people interviewed in the west asserted that: “the big people are just using corona to keep themselves in government;” and that: “… for him [President Museveni], life is okay because he has all the food and money around himself. But for us, all the ways we can utilise to make money, and getting food are closed.”

What were experienced as oppressive controls were partially lifted for periods but reinvigorated during a second lockdown in mid-2021. Threats of violence, and actual violence, were also commonplace when COVID-19 vaccines were rolled out in 2022. Indeed, it became hard to move around without a vaccination certificate, and there were numerous reports of people forgetting their vaccine certificates at home and receiving multiple unwanted vaccinations at international border crossings as a condition of entry to/from the DRC. One farmer summarised the situation by explaining that it was hard to argue with soldiers, because “these military are like untouchable human beings… our military is authority itself.”

Some people found ways of by-passing check points and socialising in secret, or by arrangement with the UPDF, LDUs and police. The formal closure of bars and night clubs was subverted. This involved closing the front of the building and encouraging people to enter the premises around the back of the building or drinking discreetly outside. Financial arrangements were made in advance with the security forces and government officials. The owner of a bar and guest house summed up the situation by saying: “the security people became important business partners to the extent of offering them drinks”; and “It has been a tough time … those who could not afford to pay [bribes] were put out of business.”

For many, especially those who were already very poor, livelihoods were seriously affected. It was observed in September 2022 that almost half of the children who had been sent home from school from March 2020 to January 2022 were still not being educated. Their parents had no money for the minimal fees. We were asked about the point of the vaccinations and if we thought COVID-19 was a real disease, or just a way for the government to enhance the power of President Museveni. An often-repeated story was that the vaccination might be a way to put something in the body so that security forces could monitor them.

These kinds of observations and discussions were discussed with officials involved in the COVID-19 National Task Force, including a senior military figure. Their response was to accept that unfortunate things probably happened, but that they were relatively unimportant. The crucial issue was to keep the disease under control in the cities, and that required restricting movement within and between urban and rural places. Some of them went on to argue that containment had been relatively successful. With respect to the experiences of rural and border populations, the term ‘collateral damage’ was not used, but the implication was clear. In contrast to Sierra Leone, embedding the military in epidemic control and health security agendas has strengthened unaccountable and authoritarian rule. The speed with which President Museveni responded to the COVID-19 pandemic in 2020 served his interests, because he was going to face considerable opposition to his re-election in the upcoming elections. Imposing the first lockdown provided an opportunity to prevent crowds gathering during the election campaign, while also helping to inject fear and limit debate (Parker et al 2020; Cheeseman 2021). The UPDF was fully complicit in these developments and have not sought to act as a guarantor of democratic elections. The President’s control of the army was such that he could effectively use it as his instrument.

Thus, during the COVID-19 pandemic, Uganda drew on previous experience with Ebola control, including the warzone of the northern part of the country in 2000/1, and the legitimization of enforcement established in the West African Ebola outbreak. COVID-19 control was a militarized project, and the overt securitization of public health was linked to reinforcing the President’s power. The approach was more aligned with China than the UK or US, even though the UPDF trains with US and UK forces. In China, the role of the military in epidemic control built on a well-established command-control system in the country that is held up by supporters of official Chinese approaches to governance as being entirely positive (Ma et al 2016). Margaret Chang, the long-term director-general of WHO, was also enthusiastic about the way China had “… done a very good job in showing responsibility to the international community…” and had “… mobilized the nation to maintain the safety of the people’s lives” (Vanderklippe 2020).

In this context, it is revealing how Uganda has been valorised by international organisations. This has occurred even though COVID-19 testing has not occurred widely, and - as is the case in Sierra Leone - national data on infections and fatalities is not comprehensive. Overall COVID-related deaths reported in Uganda by WHO are 3,632, and 26.4 million vaccine doses have been administered to a population of around 45.8 million. To the extent that it is possible to use the published Ugandan COVID-19 tracking data, declines in reported infections mostly do not correspond with the introduction of control measures. For example, using available epidemiological COVID-19 data for Uganda and South Africa collated by John Hopkins University, Laing et al (2023) have suggested that control measures might have had some influence on the first outbreak, but had little or no effect on the second and third wave. Instead, analysis of the data suggests rapid uncontrolled spread and spontaneous resolution of outbreaks.

Overall, the Ugandan results indicate that public policy was much less impactful than it was in South Africa. Nevertheless, Uganda is hailed as a ‘role model for pandemic containment’ (Sarki et al 2020). Indeed, the Lancet Commission claimed that Uganda was more effective at containing COVID-19 than any other African country, and it is ranked 10^th^ out of 191 nations worldwide (Lancet 2020). In August 2021, the Global Fund published a piece called ‘Uganda’s Remarkable Response to COVID-19’ and explained that: “Uganda achieved that feat by swiftly deploying health systems and community responses created to fight other infectious disease, including HIV, TB and malaria” (Global Fund 2021). Along similar lines, the Commonwealth Secretary General praised President Museveni for his response, claiming that he had succeeded in his efforts by: “listening to science, listening to the empirical evidence, planning and helping to get the population to support the measures” (Ajuna 2021). Recognising this ‘achievement’, the British Medical Journal and the National Health Service Health Education Program awarded President Museveni a medal for his “extraordinary leadership in pandemic management” (Kasibwe 2022).

In September 2022, there was a new Ebola outbreak in Mubende, the central region of Uganda. Military officers swiftly switched their attention to Ebola, and the response to it has been imbued with military rhetoric, as illustrated at the start of this article. In contrast to Sierra Leone, the government’s view, or at least President Museveni’s, is that Ebola is less of a threat than COVID-19, because it is not hidden. Those infected are known: “This is an enemy we can easily fight. The big battle is on washing hands,” (quoted by Kazibwe, 2022.)

Security agendas associated with the President’s authority, have dominated public discourse in Uganda. The total number of deaths reported for the recent outbreak is 55, compared to the 224 deaths during the previous outbreak in 2000/1. Containment of the outbreak has been described as a military victory, and even successful treatment of infected medics is presented to the press in an overtly militarised format. Meanwhile, in July 2023, testimonies from more than 200 people accusing Ugandan officials, including the president and his son, of torture, killings and other crimes against humanity were submitted to the International Criminal Court in The Hague (New York Times 2023). The events described occurred before and after the 2021 elections, at the same time as the lauded COVID-19 lockdowns.

## Conclusion

Developments in Uganda and Sierra Leone illustrate changes in militarised epidemic control, particularly since the 1990s, and shifts in international perceptions of state public authority in Africa. In parts of Uganda, soldiers and police were involved in violent coercion when enforcing epidemic control measures during outbreaks of Ebola (2000-2001 and 2022) and COVID-19 (2020-2022). This was largely accepted by international organisations. Indeed, the government has been valorised for its achievements. However, while focussed militarised controls may have been helpful in containing Ebola, it is much less clear that this was the case for COVID-19. At a local level, enforced constraints were experienced as oppressive, and efforts were made to subvert them. Moreover, epidemiological data suggests that while the militarised approach may have had some influence on the first wave of COVID-19, it had little or no influence on the second and third waves of infection.

Meanwhile, in Sierra Leone, violence perpetrated by soldiers during the Ebola outbreak of 2014 was linked to a Security Council Resolution and was supported by international as well as national armed forces. The degree to which soldiers limited the spread of the disease on the ground is unknown, but it is irrefutable that incidents experienced as oppression occurred in some locations. Subsequently, the army did not use its enhanced authority to prevent a transfer of political power, and generally resisted being drawn into socially alienating acts to contain COVID-19. The fact that the army acted in this way could be viewed as something to celebrate, although it seems to have been largely ignored in assessments of African responses to the pandemic. There is a paucity of data with which to make a full assessment but resulting COVID-19-related health outcomes do not appear to have been dramatically worse than Uganda. The Ugandan population is more than four times that of Sierra Leone but has 28 times more recorded COVID-related deaths, and Sierra Leone reports much higher vaccine coverage. Nevertheless, it is the militarised control model that has been foregrounded as the most viable way to co-ordinate and shape responses.

International agencies now sometimes choose to openly comply with enforcement policies and authoritarian agendas. They willingly provide funding and largely ignore human rights violations. Moreover, assessments of the approach by influential analysts have been remarkably positive, even when evidence about health outcomes is weak, and may have had counter-productive effects. From the evidence presented in this article, it was unlikely to have been the main factor linked to the ending of epidemic outbreaks. This trajectory in the public authority of formal governance has occurred for a variety of reasons, including fears about the spread of diseases to affluent countries, and has important political effects. It underlines the insights of scholars foregrounding the way in which many African governments are using the language of securitisation to foreclose debate and enhance centralised presidential authority in realms beyond public health (Fisher and Anderson, 2015). It also reflects a drift towards more open acceptance that liberal democracy may not be the best form of governance in a crisis, and that other models, such as that of China, may be more effective. It seems that militarised enforcement is a likely public authority legacy of Ebola and COVID-19, with broad implications for future disease outbreaks.

### Policy recommendations

Assessments of the positive health outcomes of militarised disease control in Uganda and Sierra Leone are not grounded in robust evidence. Assuming enforcement is efficient in public health terms is unhelpful and misleading.

Those calling for mobilization of military personnel for epidemic control should be aware of the diverse effects, including violent acts perpetrated on vulnerable people. Militarization should be a strategy of last resort.

Policy makers and public health practitioners need to recognise that, as militarised enforcement becomes more established as a norm, epidemic controls will have important effects on modes of public authority exercised by states and by international actors. They we will be complicit in those political processes.

When militaries are involved in pandemic responses, national governments and their international partners should build in oversight mechanisms to ensure that enforcement measures are appropriately monitored and constrained.
